# Anticandidal Activity of Lipopeptides Containing an LL-37-Derived Peptide Fragment KR12

**DOI:** 10.3390/molecules30071598

**Published:** 2025-04-03

**Authors:** Malgorzata Anna Paduszynska, Damian Neubauer, Wojciech Kamysz, Elzbieta Kamysz

**Affiliations:** 1 Department of Inorganic Chemistry, Faculty of Pharmacy, Medical University of Gdansk, 80-416 Gdansk, Poland; 2 Laboratory of Chemistry of Biological Macromolecules, Department of Molecular Biotechnology, Faculty of Chemistry, University of Gdansk, 80-308 Gdansk, Poland

**Keywords:** lipopeptides, antimicrobial peptides, KR-12, LL-37, fungal biofilm, *Candida*

## Abstract

Candidiasis belongs to common fungal infections and is usually mild and self-limiting. However, in patients with immunodeficiencies, it can transform into invasive infections with high mortality. Long-term antifungal treatment can lead to the emergence of resistance. The problem is further complicated by the development of fungal biofilm resistant to conventional antimicrobials. Due to a limited choice of available antifungals, the development of novel active agents, such as antimicrobial peptides (AMPs), is highly desirable. Human cathelicidin LL-37 is an intensively studied AMP with a confirmed broad spectrum of antimicrobial activities. Due to the relatively high costs of production, the design of shorter analogs of LL-37 has been recommended. In this study, we synthesized a KR12 amide, KRIVQRIKDFLR-NH_2_, and its 24 derivatives obtained by substitution with fatty acids. The compounds were tested for their antifungal potential. They exhibited activity against the *Candida albicans*, *C. glabrata*, *C. tropicalis* and *C. lipolytica*. Five compounds: C_10_-KR12-NH_2_, C_12_-KR12-NH_2_, C_14_-KR12-NH_2_, 2-butyloctanoic acid-KR12-NH_2_, and 4-phenylbenzoic acid-KR12-NH_2_ were highly active against planktonic cells. C_14_-KR12-NH_2_ demonstrated also activity against *C. albicans* biofilm cultured on polystyrene for 24, 48 and 72 h. Lipidation has proven to be an effective strategy for improving microbiological activity of the KR12-NH_2_ peptide.

## 1. Introduction

Due to a relatively limited choice of available antifungals and the emergence of fungal resistance, the effectiveness of antifungal therapy appears to be insufficient. Resistance to antifungals has frequently been reported for important fungal pathogens [1,2]. Moreover, the therapy for life-threatening fungal infections often requires the use of strong antifungals such as amphotericin B and causes numerous and severe side effects [3]. The problem is further complicated by formation of fungal biofilm which offers a mechanical and biochemical shield from the hostile environment such as antimicrobials. Microorganisms form organized 3D structures, composed of microcolonies of microbes, surrounded by water channels providing nutrients and metabolic products on the surface of biomaterials and human tissues. These sessile communities are up 1000 times more resistant to common antibiotics relative to their planktonic counterparts. Biofilm-related infections account for over 80% of chronic infections, especially those related to application of medical materials e.g., cardiac implants, catheters, and vascular and orthopedic prostheses. Due to the presence of ‘persister cells’, which can repopulate once the antibiotic has been withdrawn, such infections are often impossible to treat because they can return even after the therapy has been completed [4,5,6,7].

*Candida* species belong to common etiological factors of fungal diseases. Candidiasis infections are usually superficial, mild, and self-limiting. However, in high-risk patients with immunodeficiencies caused by medical conditions and applied therapies they can transform into invasive fungal infections with high mortality [8,9,10]. *Candida albicans* is a frequent pathogen isolated from patients suffering from candidiasis and belongs to well-known fungal biofilm formers [11]. Moreover, in recent years, infections caused by a group of pathogens known as non-albicans, *Candida* (e.g., *C. glabrata*, *C. krusei*, *C. parapsilosis*, and *C. tropicalis*) are still common [12]. Along with the increased occurrence of fungal infections related to biofilm and/or resistant strains emerges an urgent need for the development of novel antifungal strategies [13].

Endogenous antimicrobial peptides (AMPs) constitute an extremely inspiring source for the search for novel antimicrobials. The compounds are expressed by higher organisms as the first-line of defense to prevent or combat microbial infections. Human tissues and mucosal surfaces express over 100 AMPs. The compounds are produced on sites crucial for the defense against microbial infection e.g., skin, ocular surface, digestive and urinary tract, and neutrophils. AMPs exhibit both antimicrobial and immunostimulatory activities. They also possess the ability to neutralize lipopolysaccharide (LPS) and play an important role in wound-healing processes [14]. Due to the fact that the compounds are expressed by humans, a lower risk of side effects during therapy might be expected. Human cathelicidin LL-37 is an intensively studied AMP with a confirmed broad spectrum of antimicrobial activities including anti-biofilm potential [15,16]. However, in the case of this compound, its immunostimulant activities seem to be much more promising than the direct antimicrobial action that has been seen at higher concentrations. Due to the relatively high costs of production, the design of active shorter analogs of LL-37 with improved biological features has been recommended. Lipidation is considered an effective strategy to improve the microbiological properties of AMPs. Among the premises of this approach are the biological activities of bacterial lipopeptides such as surfactins, iturins, and fengicins. The compounds are widely studied due to their multiple potential applications in the fields of medicine, environmental protection, and industry [17]. The introduction of fatty acid residues to the amino acid sequences of AMPs not only can increase their antimicrobial potential, but also might improve the stability of compounds [18,19,20].

In this study, we investigated lipopeptides containing the shortest α-helical fragment of LL-37 with documented antimicrobial activity, the KR12 amide (KRIVQRIKDFLR-NH_2_) [21,22] combined with linear and aromatic fatty acids. The peptides used in this study are listed in Table 1.

Lipopeptides can be divided into two main groups of KR12-NH_2_ (compound **X**) analogs: (1) those with an aliphatic carboxylic acid conjugated to (1A) the N^α^-terminal amino group of KR12-NH_2_ (peptides **I**–**VII**; **XII**, **XVIII**–**XX**), (1B) the N^ε^-amino group of lysine (**XI**, **XIII**–**XV**, **XVII**), or (1C) both types of amino groups (**XVI**); and (2) analogs with aromatic carboxylic acid (**VIII**, **IX**, and **XXI**–**XXV**). Aliphatic carboxylic acids are of different chain lengths (C_2_–C_14_) and some of them are branched (2-ethylhexanoic acid, 2-butyloctanoic acid). Similarly, aromatic carboxylic acids have different numbers of methylene entities between the phenyl ring and carboxylic group (from benzoic acid with 0 to 4-phenylbutanoic acid with 3) and different degrees of saturation (i.e., 3-phenylpropionic acid with a single bond, *trans*-cinnamic acid with a double bond, and phenylpropiolic acid with a triple bond). Moreover, lysine was modified with octanoic acid in the parent molecule (**IV**, **XI**, **XVI**), in its retro analog (**XVII**, **XVIII**). Some residues were substituted with such lysine derivative (**XV**) or it was added on the N- or C-terminus (**XII**–**XIV**). Octanoic acid was selected for such modifications based on previous results that provide evidence of optimum selectivity between antimicrobial and hemolytic activity for this particular acid residue [21]. Such a wide spectrum of carboxylic acids types and lengths, and different C_8_ positions gives an opportunity to link antifungal activity with specific structural characteristics of peptides.

The compounds were initially designed as potential antibacterial agents and some of them proved to be effective against ESKAPE pathogens and promising antibiofilm agents against *Staphylococcus aureus* [21,22]. The aim of the current study was to investigate the group of KR-12-based lipopeptides according to their anticandidal activity against planktonic cells of different *Candida* species. The most effective agents were also tested against biofilms formed by *Candida albicans*.

## 2. Results

### 2.1. Activity of Peptides and Reference Compounds Against Planktonic Cells

**Figure 1:**
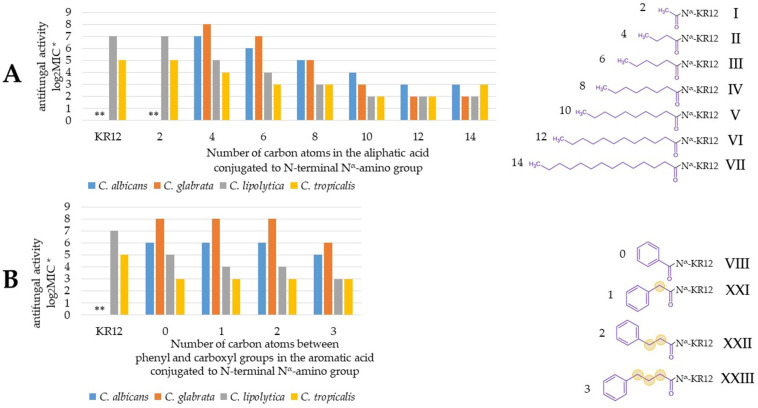
Log_2_MIC vs. (**A**) the number of carbon atoms in the chain of aliphatic carboxylic acid conjugated to the N-terminal amino group; (**B**) the number of carbon atoms between the phenyl and carboxyl groups of conjugated aromatic acid residue. * log_2_MIC is applied for readability ** The MICs of X (KR12) and peptide I against *C. albicans* and *C. glabrata* were above the applied peptide concentrations, therefore, no bars are shown.

The antifungal activity presented by the tested peptides varied depending mostly on their structure. However, certain differences in the sensitivity of various *Candida* species to individual compounds have also been noticed (Table 2). In general, *C. lipolytica* and *C. tropicalis* were more susceptible to the compounds as compared to the other strains. The peptides without a fatty acid attached, KR12-NH_2_ (**X**) and its acetylated derivative (**I**), did not inhibit the growth of *C. albicans* and *C. tropicalis* once applied at the highest concentrations (256 mg/L). Both strains were also resistant to the lipopeptide containing two residues of octanoic acid (**XVI**). The highest antifungal activity was exhibited by derivatives with linear unbranched fatty acids (**II**–**VII**). The optimum activity was achieved by the attachment of longer chains, dodecanoic (**VI**), and tetradecanoic acid (**VII**). On the basis of the results obtained for the tested strains (MIC = 4–8 mg/L), the compounds can be classified as highly active antimicrobial peptides [23]. In general, peptides containing octanoic acid (**IV**, **XI**–**XVIII**) had significantly lower anticandidal activities, especially against *C. albicans* and *C. glabrata* (MIC = 64–128 mg/L). Attachment of branched-chain fatty acids (**XIX**, **XX**) and aromatic fatty acid chains (**VIII**, **IX**, **XXI**–**XXV**) to the KR12-NH_2_ allowed us to obtain peptides with a moderate antifungal activity. However, two compounds belonging to this group, 2-butyloctanoic acid-KR12-NH_2_ (**XIX**) and 4-phenylbenzoic acid-KR12-NH_2_ (**XXV**), presented high antifungal activity (MIC 4–16 mg/L). The type of bond between the carboxyl group and the phenyl ring in aromatic acids appears to be optimal in the case of *trans*-cinnamic acid (**IX**, double bond), but the differences in MIC are most pronounced in the case of *C. glabrata* (64 vs. 128 and 256 mg/L for phenylpropiolic and phenylpropionic acid, respectively). It can be seen that the antifungal activity gradually increases with carboxylic acid chain elongation, both aliphatic and aromatic (Figure 1). Activity was presented as log_2_MIC (base 2 logarithm of MIC) to ease comparison between compounds (e.g., 8, 7, 6 instead of 256, 128, 64 μg/mL). Analogously to MIC, lower log_2_MIC values indicate higher antifungal activity.

In the case of analogs with aliphatic acids, the most potent antifungal compounds are modified with C_10_–C_14_ fatty acids (**V**–**VII**), whereas among analogs with aromatic acids, the lowest MIC was recorded for **XXIV**, which has the longest chain between the phenyl and carboxyl groups. Moreover, increasing the number of phenyl rings in conjugated acids (**VIII** and **XXV**) improved antifungal activity. This is presumably due to the increasing size and hydrophobicity of the lipophilic fragment (analogously to extending alkyl chain length and decreasing MIC; Figure 1). The results show that the type of amino group being acylated (N^α^ or N^ε^) plays a key role in antifungal activity. Conjugation of octanoic acid to the N^α^ amino group resulted in more potent antifungal peptides than that of N^ε^ modified (**IV** vs. **XI** and **XII** vs. **XIII**) but the effect usually was not so pronounced (one dilution). Another aspect that is associated with the antifungal activity of peptides is electrostatic charge (number of positively and negatively charged residues) [24]. Nevertheless, pairs of peptides, such as **IV** and **XII**, **XI** and **XIII**, or **XIV** and **XV**, despite the difference in the net charge (+4 vs. +5, +4 vs. +5, and +4 vs. +3, respectively) only in some cases is there a slight effect on antifungal activity. Some studies on retro-analogs have shown that reversing the amino acid sequence of peptides can enhance their antimicrobial activity [25,26] However, retro-analogs of this study mostly were less active than the parent molecules (**XI** vs. **XVII**, **IV** vs. **XVIII**). The literature provides evidence that the structure of the carboxylic acid attached to the peptide significantly affects biological activity [27]. The diversity of carboxylic acids used in this research includes both straight-chain and branched ones. What can be noticed is the diminished antifungal activity against *C. albicans* and *C. glabrata* in the case of **XIX** and **XX** as compared to that of analogs with straight-chain acids with an identical number of carbon atoms (**VI** and **IV**, respectively).

It is worth noting that almost all the peptides exhibited fungicidal activity (Table 2). The MFCs were equal to or twice as high as the corresponding MICs. The fungicidal activity against all *Candida* strains was presented also by reference compounds: benzalkonium chloride and nystatin. Thus, fluconazole exhibited fungicidal activity against *C. albicans* and *C. glabrata*. All the reference antifungals inhibited microbial growth once applied at concentrations lower than that of the most active peptides (MIC = 0.5–4 mg/L).

### 2.2. Activity of the Peptides and Reference Compounds Against Candida Biofilm

The MBECs (Table 3) were determined for reference antifungals, KR-12-NH_12_ and its derivatives which have proven to be the most effective against planktonic cells (C_10_-KR12-NH_2_, C_12_-KR12-NH_2_, C_14_-KR12-NH_2,_ 2-butyloctanoic acid-KR12-NH_2_, and 4-phenylbenzoic acid-KR12-NH_2_). Biofilms of different maturity were resistant to almost all the peptides at the tested concentrations. C_10_-KR12-NH_2_, 2-butyloctanoic acid-KR12-NH_2_, and 4-phenylbenzoic acid-KR12-NH_2_ did not exhibit antibiofilm activity against any type of biofilm. The same results were obtained for fluconazole. Despite high activity against planktonic cells, fluconazole showed no antibiofilm activity even when applied once at the highest concentration. C_12_-KR12-NH_2_ demonstrated activity against biofilm cultured for 24 h at the highest concentration (512 mg/L). Among the peptides, only the C_14_-KR12-NH_2_ showed some promising results. The compound demonstrated equal activity to that of nystatin against structures formed by *Candida albicans* for 24 and 48 h (MBEC = 256 mg/L), while MBEC for 72 h biofilm was twice as high. The obtained MBECs were significantly higher as compared to those of the corresponding MICs and MFCs. The most effective compound was benzalkonium chloride. However, the concentrations effective against 24 and 48–72 h biofilms were, respectively, over 30 and 60 times higher than those of MIC.

### 2.3. Visualization of Candida Albicans Cells Treated with Lipopeptide C_14_-KR12-NH_2_ by Fluorescence Microscopy

Fluorescence microscopy was applied to visualize the influence of selected lipopeptide C_14_-KR12-NH_2_, which has displayed the strongest anticandidal activities in MIC, MFC, and MBEC assays, on *Candida albicans* cells. Benzalkonium chloride was used as a conventional membrane-disrupting agent (positive control). SYTO 9 and propidium iodide (PI) were applied to stain the components of fungal cells. SYTO 9 is a green fluorescent dye that stains both live and dead cells. PI does not cross intact plasma membranes, but once it enters the cell, it binds and emits red fluorescence [28].

**Figure 2:**
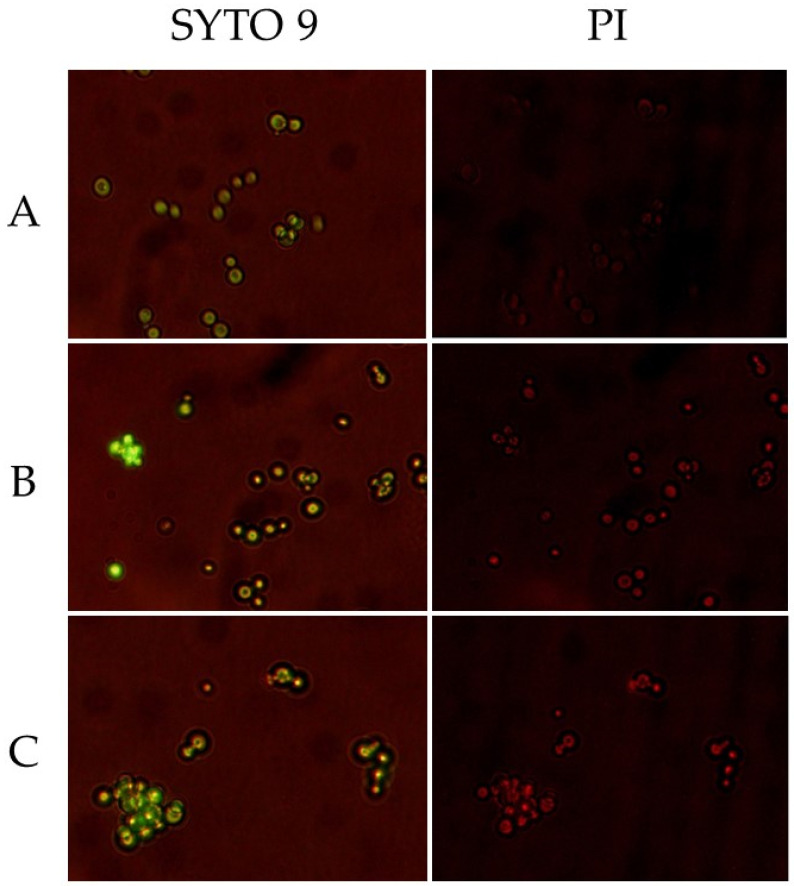
Fluorescence microscopy images of *C. albicans* ATCC 10231. (**A**)—untreated cells (negative control); (**B**)—cells exposed to benzalkonium chloride (positive control); (**C**)—cells exposed to lipopeptide C_14_-KR12-NH_2_.

For negative control we have observed a faint green fluorescence (SYTO 9) and an absence of red fluorescence (PI) inside the fungal cell (Figure 2). The cells treated with lipopeptide and benzalkonium chloride show both green and red fluorescence. The compounds permeabilize cell membranes, which allows PI to penetrate and bind to double-stranded DNA.

### 2.4. Plasma Stability

**Figure 3:**
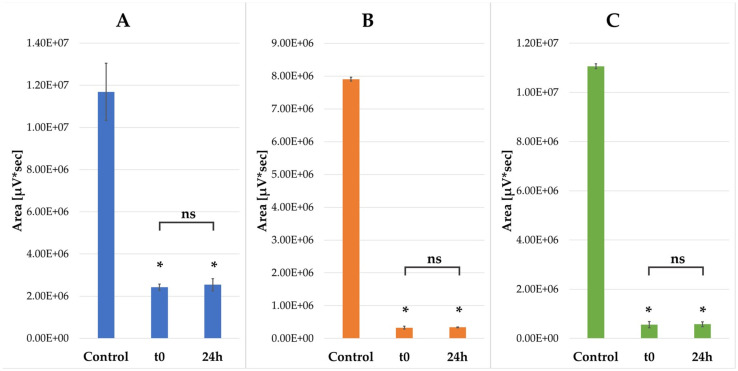
Plasma stability of selected peptides. Peak area of peptide on chromatogram: control—peptide in RPMI medium without serum, t0—peptide with serum at the beginning of the experiment, 24 h—peptide after 24 h incubation with plasma. *—*p* < 0.001, ns—not significant. (**A**) Peptide **VII**, (**B**) Peptide **XIX**, (**C**) Peptide **XXV**.

Compounds **VII**, **XIX**, and **XXV** were selected for stability studies due to their exceptional antifungal properties. Stability was tested after 24 h incubation at 37 °C using LC-MS. The retention time of peptide **VII** was the same as that of compounds present in plasma alone. Therefore, absorbance at 214 nm could not be used in this case. To study VII degradation ESI-MS was used ([M+2H]^2+^ ion of *m*/*z* 891.10). The stability of the remaining compounds was evaluated using a photodiode array detector set at 214 nm. The results of the plasma stability test are presented in Figure 3.

The starting concentration of each peptide was identical, both within the control and samples supplemented with plasma. The differences between the control and t0 (also 24 h) are undoubtedly significant (*p* < 0.0001). It can therefore be concluded that a major portion of each peptide was bound to plasma proteins from the beginning of the experiment. This is not surprising since albumins can strongly bind drugs, including peptides [29]. Moreover, human serum albumins have binding sites dedicated to fatty acids [30]. Nevertheless, it seems that there is no significant degradation of examined peptides even after 24 h of incubation with plasma.

## 3. Discussion

Lipidation has proven to be an effective strategy for improving the microbiological properties of the KR12-NH_2_ peptide. Previous studies revealed that the introduction of a fatty acid chain allows us to obtain peptides with promising antibacterial activities [21,22]. The influence of the structure of substituted fatty acids as well as the lipidation pattern was noticed. Among derivatives containing linear fatty acid residues, an analog with octanoic acid turned out to be the most promising antibacterial agent [21]. The C_8_-KR12-NH_2_ peptide (compound IV) inhibited the growth of both Gram-positive and Gram-negative bacteria belonging to the ESKAPE pathogens and can be classified as a highly active antibacterial peptide [21,23]. Moreover, the compound revealed strong antibiofilm activity against *Staphylococcus aures* (MBEC 4–16 mg/L). In another study, an attempt has been made to further optimize the biological activities of the compound IV by the conjugation of octanoic acid to the N^α^-terminal amino group of KR-12, N^ε^-amino group of lysine of KR12-NH_2_ in a parent molecule (**IV**, **XI**, **XVII**), in its retro analog (**XVII**, **XVIII**), substitution with such lysine derivative (**XV**), or its addition on the N- or C-terminus (**XII**–**XIV**). None of those modifications improved antibacterial activities. The obtained compounds showed a lower antibacterial activity as compared to that of C_8_-KR12-NH_2_. Similarly, as in the current study, the conjugation of KR12-NH_2_ with two C8 entities resulted in a total loss of antimicrobial activity [22]. In our study, the C_8_-KR12-NH_2_ presented antifungal activity lower than that of the derivatives containing longer-chain acids. Similarly, as previously further modifications of C_8_-KR12-NH_2_ did not improve its antifungal potential. The most promising compounds among the linear fatty acid derivatives tested against *Candida* strains were C_12_-KR12-NH_2_ and C_14_-KR12-NH_2_. They also showed some antibiofilm activities against biofilm formed by *Candida albicans*, as well as by *S. aureus* in the previous study (MBEC 64–256 mg/L) [21]. The obtained results suggest that the antifungal activity of compounds with linear unbranched fatty acid increases with the length of the fatty acid chain (Table 1, Table 2 and Table 3). The strongest antifungal potential was presented by the lipopeptide containing tetradecanoic fatty acid. Further optimization of structures towards enhancement of antifungal potential should include attachment of longer-chain fatty acids.

Interestingly, among peptides containing cyclic and branched fatty acids, the same compounds demonstrated the highest antimicrobial and anticandidal activities. The 2-butyloctanoic acid-KR12-NH_2_ and 4-phenylbenzoic acid-KR12-NH_2_ demonstrated a broad spectrum of antimicrobial activity against ESKAPE pathogens and *Candida* strains [22]. Unfortunately, despite high activity towards planktonic fungal cells, they did not reveal antibiofilm potential. A similar lack of activity against *C. albicans* biofilm was found for peptide C_10_-KR12-NH_2_, which showed promising results against staphylococcal biofilms [21]. Generally, such significant differences between the activity against planktonic cells as compared to that against biofilm have often been seen for conventional antimicrobials [31,32]. In the present study, the biofilms formed by *C. albicans* turned out to be resistant to fluconazole applied at the whole concentration range. *Candida albicans* is the most common etiological factor of biofilm-associated fungal infections [33,34]. Structures formed by this species are well known for their resistance to antimicrobials including nystatine and fluconazole [35,36]. Fluconazole acts via inhibition of ergosterol synthesis and is recommended for oral and parenteral use in fungal infections as a first-line therapy. Unfortunately, due to the fungal resistance and the development of biofilm, its clinical effectiveness declines [37,38]. In our study, nystatine showed some antibiofilm activity, but only at a concentration of 256 mg/L which was over 100 times higher than the determined MIC. Also, benzalkonium chloride and C_14_-KR12-NH_2_ exhibited activity against biofilm at relatively high concentrations.

It is worth noting that a satisfying antifungal activity of the lipopeptide occurred at a concentration much higher than that determined as toxic to human keratinocytes and erythrocytes (Table 2 and Table 3) [21]. The lack of selectivity to fungal cells is the result of the membrane-related mechanism of action. The lipopeptides act by disrupting fungal membrane permeability [39]. The ability to permeabilize fungal cell membranes was visualized with fluorescence microscopy for the lipopeptide C_14_-KR12-NH_2_, which is consistent with its anticandidal and hemolytic activities. The results obtained previously on human cells suggest that, due to expected potential side effects, only topical application of peptides could be considered [21,22]. However, application of nystatin and benzalkonium is also limited to topical therapy of infections. In general, due to the similarities of fungi and human cells, the toxicity of antifungals significantly limits the scope of available active agents. Considering the growth of the population of high-risk patients and the fact that long-term antifungal prophylaxis and treatment lead to the emergence of antifungal resistance, the increase in therapeutic difficulties of *Candida* infections is evident [40,41]. Considering the obtained results, lipidation of AMP seems to be a promising approach to the development of new effective antifungals. The introduction of fatty acid residues to KR12 peptide allowed us to obtain microbiologically active molecules with satisfying stability in human plasma. However further work on optimization of structures as well as on application of appropriate carriers for peptides to improve the biological properties is required.

## 4. Materials and Methods

### 4.1. Fungal Strains and Culture Conditions

Fungal strains were purchased from the Polish Collection of Microorganisms (Polish Academy of Science, Wroclaw, Poland). Four reference strains of *Candida* (*C. albicans* ATCC 10231, *C. glabrata* ATCC 15126, *C. lypolytica* PCM 2680, and *C. tropicalis* PCM 2680) were cultured on the Dextrose Sabouraud Agar (DSA, Neogen, Lansing, MI, USA). Before performing the assays, the fungi were cultured in a Sabouraud dextrose broth (DSB, Neogen, Lansing, MI, USA) for 48 h under aerobic conditions at 25 °C. Afterward, the fungal cultures were centrifuged (2500 rpm, 10 min) and resuspended in the appropriate liquid medium for appropriate inoculums for the performed assays.

### 4.2. Antimicrobials

The following reference antifungals were applied: benzalkonium chloride, fluconazole (Sigma-Aldrich, Darmstadt, Germany), and nystatin (Fargon, Krakow, Poland).

Peptide KR12-NH_2_ (X) and all its derivatives (I–IX and XI–XXVI) were synthesized by the solid-phase method using 9-fluorenylmethoxycarbonyl (Fmoc) chemistry on a resin modified by a Rink amide linker with loading 1.0 mmol/g (Orpegen Peptide Chemicals GmbH, Heidelberg, Germany) according to procedures described previously [13,14]. Couplings were performed using DIC (*N*,*N*’-diisopropylcarbodiimide, Peptideweb, Zblewo, Poland) and OxymaPure (ethyl(2*E*)-cyano(hydroxyimino)acetate, Iris Biotech GmbH, Marktredwitz, Germany) in a 3-fold molar excess in DMF (N,N-dimethylformamide, Sigma-Aldrich, Poznan, Poland). Fmoc deprotection was achieved using 20% piperidine (Iris Biotech GmbH, Marktredwitz, Germany) in DMF. All peptides were removed from the resin along with the side chain deprotection in a one-step procedure using a mixture of trifluoroacetic acid (TFA; Apollo Scientific, Denton, UK), triisopropylsilane (TIS; Sigma-Aldrich, St. Louise, MO, USA), and water (95:2.5:2.5 *v*/*v*/*v*) [21]. Finally, the peptides were purified by solid-phase extraction (SPE) on Isolute ^TM^ SPE columns (flash, C_18_, 25 mL) (XI–XXVI) [22,42] or using the RP-HPLC on a Knauer system controlled by an LPchrom data system (Lipopharm.pl, Zblewo, Poland) with a Knauer Kromasil C8 column (8 × 250 mm, 100 Å pore size, 5 μm particle size) (I–X) [21].

The purity of the peptides was at least 95%, as determined by analytical reversed-phase high-performance liquid chromatography (RP-HPLC). Their identity was confirmed by electrospray ionization mass spectrometry (ESI-MS) [21,22].

### 4.3. Activity of Lipopeptides and Reference Antifungals Against Planktonic Fungal Cells: Minimum Inhibitory Concentration (MIC) and Minimum Fungicidal Concentration (MFC) Assays

The strains cultured described in Section 4.1 were suspended in the Roswell Park Memorial Institute 1640 Medium (RPMI, Sigma-Aldrich, St. Louis, MO, USA) at an initial inoculum of ca. 5 × 10^3^ CFU/mL. The stock solutions (1024 mg/L) of tested compounds in RPMI were added to 96-well polystyrene plates and diluted serially to well no. 10, leaving wells 11 and 12 for fertility and sterility controls. The tested *Candida* strains were exposed to graded concentrations (range 0.5–256 mg/L) of the lipopeptides and conventional antifungals (benzalkonium chloride, fluconazole, and nystatin) for 48 h under aerobic conditions at 25 °C. After incubation, the results were read visually. The MIC was taken as the lowest concentration of the compound which application resulted in inhibition of fungal growth.

To determine MFC, the wells containing MIC and two following higher concentrations were seeded on the DSA and incubated for 48 h at 25 °C. Then the results were read visually. Both MIC as well as the MFC assays were performed threetimes on three different days.

### 4.4. Activity of Lipopeptides and Reference Antifungals Against Biofilms Formed by Candida Albicans on Polystyrene 96-Well Plates: Minimum Biofilm Eradication Concentration (MBEC) Assay

Fungal suspensions in DSB were added to the wells at initial inoculums of ca. 5 × 10^6^ CFU/mL and incubated under aerobic conditions, at 25 °C for 24 h, 48 h, and 72 h, in order to grow candidal biofilms of different maturity. After the appropriate time of incubation, the wells were washed with phosphoric buffered (PBS), and fresh RPMI medium supplemented with antifungals and the lipopeptides at graded concentrations (range 16–512 mg/mL) were added. The fungal biofilms were exposed to the compounds for 24 h (aerobic conditions, 25 °C). After the exposure to antifungals, the content of the wells was aspirated and a 0.01% solution of resazurin (Sigma-Aldrich, St. Louis, MO, USA) in DSB was added. Resazurin (blue) was applied as a cell viability reagent, which upon contact with living cells is reduced to a pink resorufin. The samples with the dye were incubated for 1 h at 25 °C and the absorbance was measured at 570 and 600 nm with a microplate reader (Thermo Fisher Scientific, Waltham, MA, USA). The assays were performed four times on four different days. The results are presented as the means of the measured percentage of metabolic activity (MA) of fungi in the well when compared with positive (sample with *Candida* in DSB) and negative controls (pure DSB), which were taken as 100% and 0%, respectively. The MA of *Candida* in the samples was measured according to the following formula:

MA(%) = (ΔAbs of sample − ΔAbs of negative control)/(ΔAbs of positive control − ΔAbs of negative control);(1)

ΔAbs = absorbance at 570 nm − absorbance at 600 nm.(2)

The MBEC was taken as the lowest concentration of antifungal which allowed us to reduce the metabolism of fungal cells to 10% (or less) of the positive control.

### 4.5. Fluorescence Microscopy

A liquid culture of *Candida albicans* ATCC 10231 was prepared as described in Section 4.1. The fungal cells were centrifuged (2500 rpm, 10 min), washed two times with phosforic buffer (PBS), and resuspended in PBS to obtain inoculum of 0.5 × 10^6^ CFU/mL. The suspension of fungal cells was subsequently treated with the peptide C_14_-KR12-NH_2_ and benzalkonium chloride at 2 × MIC. The samples were stained with SYTO 9 Dye (Thermo Fischer Scientific, Waltham, MA, USA; final concentration approx. 7 μM) and propidium iodide (PI; Thermo Fischer Scientific, Waltham, MA, USA; final concentration approx. 40 μM) and visualized with a Delta Optical Evolution 100 microscope equipped with an epifluorescence module (Delta Optical**,** Gdańsk, Poland) with FITC and rhodamine filters, respectively.

### 4.6. Stability of Peptides

The stability of peptides C_14_-KR12-NH_2_, 2-butyloctanoic acid-KR12-NH_2_, and 4-phenylbenzoic acid-KR12-NH_2_ was tested in human plasma obtained from healthy volunteers according to the modified Jenssen and Aspmo protocol [43]. Compounds at a concentration of 50 µg/mL were incubated in 25% (*v*/*v*) human plasma solution in RPMI at 37 ± 0.1 °C. After 0 and 24 h of incubation, 100 µL of samples were added to 200 µL of 96% ethanol (Pure P.A., POCH, Avantor Performance Materials S.A, Gliwice, Poland) in order to precipitate serum proteins. Samples with human plasma (25% (*v*/*v*) solution in RPMI) and samples with solutions of peptides in RPMI (50 µg/mL) served as controls. All the samples were prepared in triplicate. The samples were incubated at 4 °C for 15 min and centrifuged (4 °C, 18,000× *g*, 5 min; Sorvall ST 16R Centrifuge, Thermo Scientific, Osterode am Harz, Germany). Subsequently, 30 µL of supernatant was transferred to a glass vial and analyzed using LC-MS (Waters Alliance e2695 system with a Waters 2998 PDA Detector and an Acquity QDa Detector—ESI-MS, software-Empower 3, Waters, Milford, MA, USA). The mobile phase contained deionized water and acetonitrile (gradient grade; POCH, Avantor, Gliwice, Poland) both supplemented with a 0.1% (*v*/*v*) formic acid (Sigma-Aldrich, Steinheim, Germany). The analyses were carried out on a HALO C18 column (4.6 × 100 mm, 2.7 μm particle size, 90 Å pore size). An ESI-MS detector was used in positive ion mode. In the case of peptide VII, ion [M+2H]^2+^ was used for detection. Peptides XIX and XXV were analyzed at 214 nm. Peptides were eluted with a 1–100% gradient of acetonitrile in deionized water over 10 min using a flow rate of 0.5 mL/min. Each peptide was analyzed in triplicate. One-way ANOVA and post-hoc Tukey’s HSD test were used for statistical analysis.

## Figures and Tables

**Table 1:** The synthesized compounds.

Compound No.	Peptide	Sequence
I	Ac-KR12-NH_2_	Ac-KRIVQRIKDFLR-NH_2_
II	C_4_-KR12-NH_2_	C_4_-KRIVQRIKDFLR-NH_2_
III	C_6_-KR12-NH_2_	C_6_-KRIVQRIKDFLR-NH_2_
IV	C_8_-KR12-NH_2_	C_8_-KRIVQRIKDFLR-NH_2_
V	C_10_-KR12-NH_2_	C_10_-KRIVQRIKDFLR-NH_2_
VI	C_12_-KR12-NH_2_	C_12_-KRIVQRIKDFLR-NH_2_
VII	C_14_-KR12-NH_2_	C_14_-KRIVQRIKDFLR-NH_2_
VIII	Benzoic acid-KR12-NH_2_	Benzoic acid-KRIVQRIKDFLR-NH_2_
IX	*trans*-cinnamic acid-KR12-NH_2_	*trans*-cinnamic acid-KRIVQRIKDFLR-NH_2_
X	KR12-NH_2_	KRIVQRIKDFLR-NH_2_
XI	C_8_^ε^-KR12-NH_2_	K(C_8_)RIVQRIKDFLR-NH_2_
XII	C_8_^α^-Lys-KR12-NH_2_	C_8_-KKRIVQRIKDFLR-NH_2_
XIII	C_8_^ε^-Lys-KR12-NH_2_	K(C_8_)KRIVQRIKDFLR-NH_2_
XIV	KR12-Lys^ε^(C_8_)-NH_2_	KRIVQRIKDFLRK(C_8_)-NH_2_
XV	[Lys^ε^(C_8_)]^12^KR12-NH_2_	KRIVQRIKDFLK(C_8_)-NH_2_
XVI	C_8_^α^,C_8_^ε^-KR12-NH_2_	C_8_-K(C_8_)RIVQRIKDFLR-NH_2_
XVII	Retro-KR12-C_8_^ε^-NH_2_	RLFDKIRQVIRK(C_8_)-NH_2_
XVIII	C_8_^α^-retro-KR12-NH_2_	C_8_-RLFDKIRQVIRK-NH_2_
XIX	2-Butyloctanoic acid-KR12-NH_2_	CH_3_-(CH_2_)_5_-CH(C_4_H_9_)-CO-KR12-NH_2_
XX	2-Ethylhexanoic acid-KR12-NH_2_	CH_3_-(CH_2_)_3_-CH(C_2_H_5_)-CO-KR12-NH_2_
XXI	Phenylacetic acid-KR12-NH_2_	C_6_H_5_-CH_2_-CO-KR12-NH_2_
XXII	3-Phenylpropionic acid-KR12-NH_2_	C_6_H_5_-CH_2_-CH_2_-CO-KR12-NH_2_
XXIII	4-Phenylbutanoic acid-KR12-NH_2_	C_6_H_5_-(CH_2_)_3_-CO-KR12-NH_2_
XXIV	Phenylpropiolic acid-KR12-NH_2_	C_6_H_5_-C≡C-CO-KR12-NH_2_
XXV	4-Phenylbenzoic acid-KR12-NH_2_	C_6_H_5_-C_6_H_4_-CO-KR12-NH_2_

**Table 2:** Minimum inhibitory concentrations (MIC) and minimum fungicidal concentrations (MFC) determined for peptides and reference antifungals [g/L]. The highest antimicrobial activity is marked in green, medium values in yellow, while the weakest antifungal activity is marked in red. Minimum hemolytic concentration (MHC_5%_—causing hemolysis of 5% of human red blood cells) determined in previous studies [13,14]. The weakest/lack of hemolytic activity is marked in green, while strong hemolytic activity is marked in red.

Compound	*C. albicans*		*C. gtlabrata*		*C. lipolytica*		*C. tropicalis*		MHC_5%_
**I**	>256 *	>256 *	>256 *	>256 *	128	256	32	64	>256 *
**II**	128	256	256	>256 *	32	64	16	16	>256 *
**III**	64	128	128	256	16	32	8	16	256
**IV**	32	64	32	64	8	16	8	8	64
**V**	16	32	8	8	4	8	4	4	4
**VI**	8	16	4	4	4	4	4	4	4
**VII**	8	8	4	4	4	4	8	8	4
**VIII**	64	64	256	256	32	32	8	16	>256 *
**IX**	32	64	64	64	8	16	8	8	128
**X**	>256 *	>256 *	>256 *	>256 *	128	128	32	32	>256 *
**XI**	64	128	>256 *	>256 *	32	32	16	32	>256 *
**XII**	64	128	128	128	8	16	8	16	256
**XIII**	128	256	256	256	16	32	16	32	256
**XIV**	128	256	128	128	16	32	16	16	>256 *
**XV**	128	128	128	256	32	32	16	16	>256 *
**XVI**	>256 *	>256 *	>256 *	>256 *	16	>64 *	16	32	>256 *
**XVII**	128	256	>256 *	>256 *	32	128	32	32	256
**XVIII**	64	128	128	256	16	32	16	16	>256 *
**XIX**	16	32	8	8	4	8	4	8	16
**XX**	32	64	128	128	8	32	8	8	256
**XXI**	64	64	256	256	16	16	8	16	256
**XXII**	64	64	256	256	16	16	8	16	256
**XXIII**	32	64	64	64	8	16	8	16	128
**XXIV**	32	64	128	128	16	16	8	16	256
**XXV**	16	32	16	16	8	16	4	4	32
Benzalkonim chloride	2	4	1	2	1	2	0.5	2	nd
Fluconazole	4	8	0.5	2	2	8	2	8	nd
Nystatin	2	8	2	2	2	8	2	4	nd

* No antimicrobial/hemolytic activity was found at the concentrations applied; nd—not determined.

**Table 3:** Minimum biofilm eradication concentration [mg/L] determined against *C. albicans* biofilms cultured on polystyrene for 24, 48, and 72 h. The highest antibiofilm activity is marked in green, medium values in yellow, while a lack of antibiofilm activity is marked in red.

Compound	MBEC 24 h	MBEC 48 h	MBEC 72 h
**X**	>512 *	>512 *	>512 *
**V**	>512 *	>512 *	>512 *
**VI**	512	>512 *	>512 *
**VII**	256	256	512
**XIX**	>512 *	>512 *	>512 *
**XXV**	>512 *	>512 *	>512 *
Benzalkonium chloride	64	128	128
Fluconazole	>512 *	>512 *	>512 *
Nystatin	256	256	256

* No antimicrobial activity was found at the concentrations applied.

## Data Availability

Data is contained within the article and Supplementary Materials.

## Supplementary Materials

The following supporting information can be downloaded at: https://www.mdpi.com/article/10.3390/molecules30071598/s1. Table S1: Chemical strucutres of the peptides.
