# Supplementary material for: Anticandidal Activity of Lipopeptides Containing an LL-37-Derived Peptide Fragment KR12
Source: Molecules. 2025 Apr 3;30(7):1598. doi: 10.3390/molecules30071598 (PMC11990879; doi:10.3390/molecules30071598)
Supplement: Supplementary file 1 [file molecules-30-01598-s001.zip › molecules-3515687-supplementary.pdf]

Table S1. Chemical strucutres of the peptides.

**Compound no.**	**Peptide**	**Structure**
**I**	Ac-KR12-NH_2	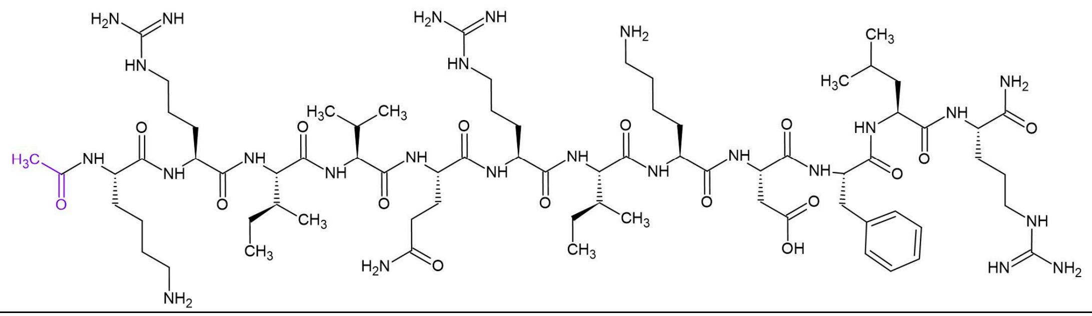
**II**	C_4-KR12-NH_2	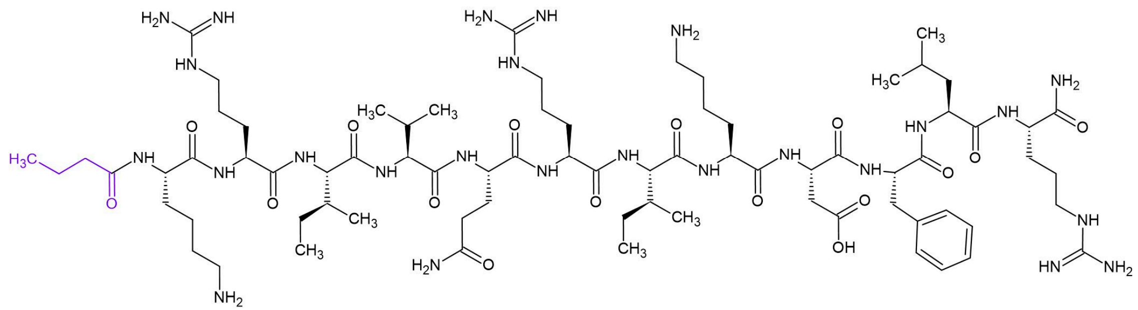
**III**	C_6-KR12-NH_2	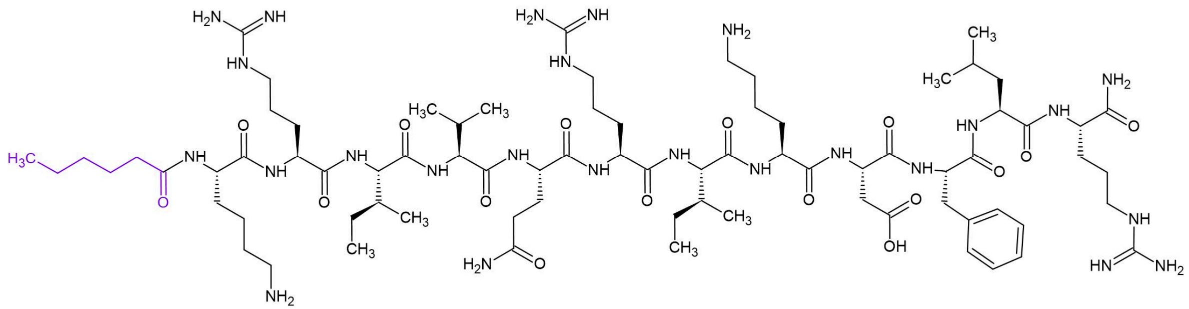

IV	C_8-KR12-NH_2	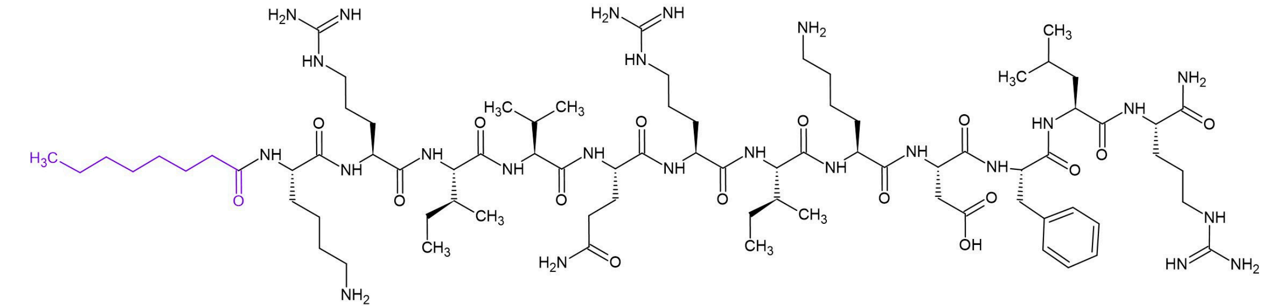
V	C_{10}-KR12-NH_2	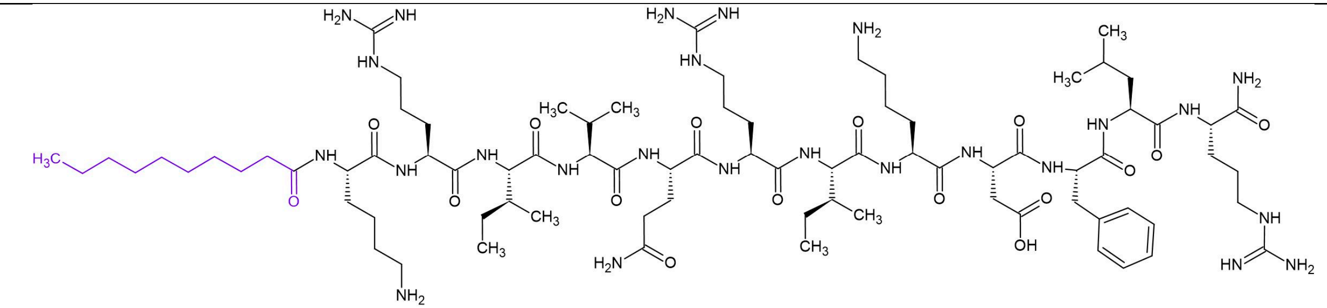
VI	C_{12}-KR12-NH_2	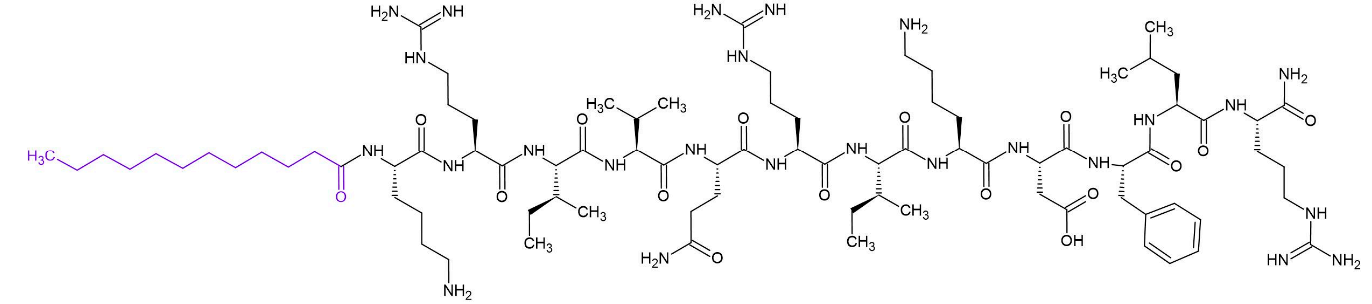
VII	C_{14}-KR12-NH_2	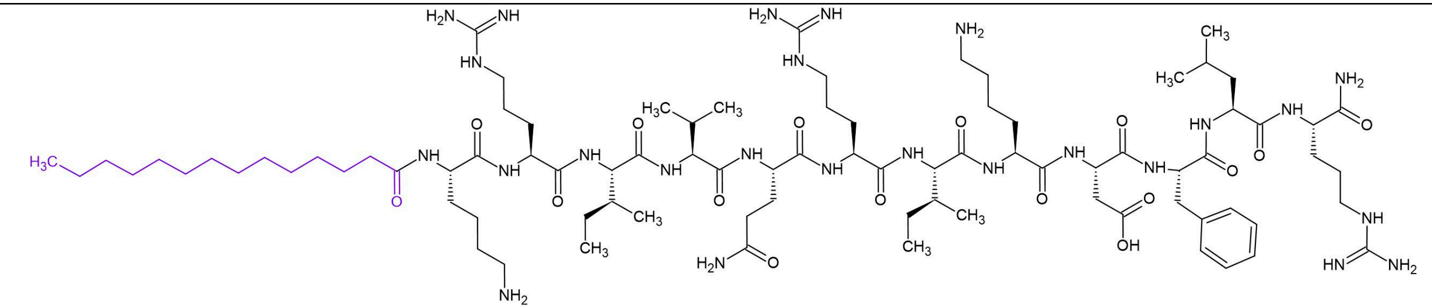

VIII	Benzoic acid-KR12-NH_2	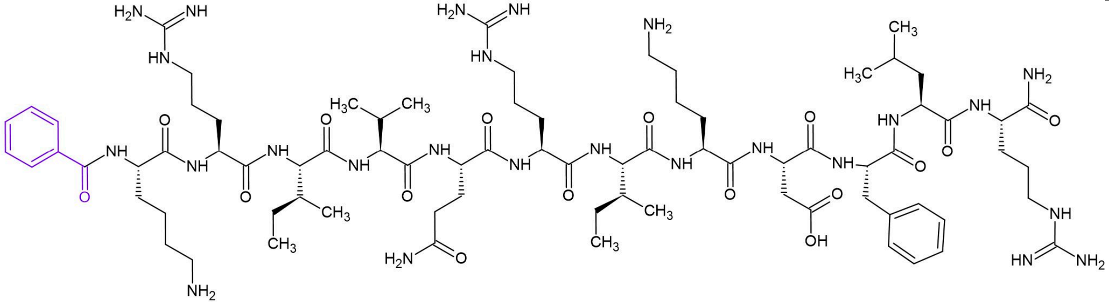
IX	trans-cinnamic acid-KR12-NH_2	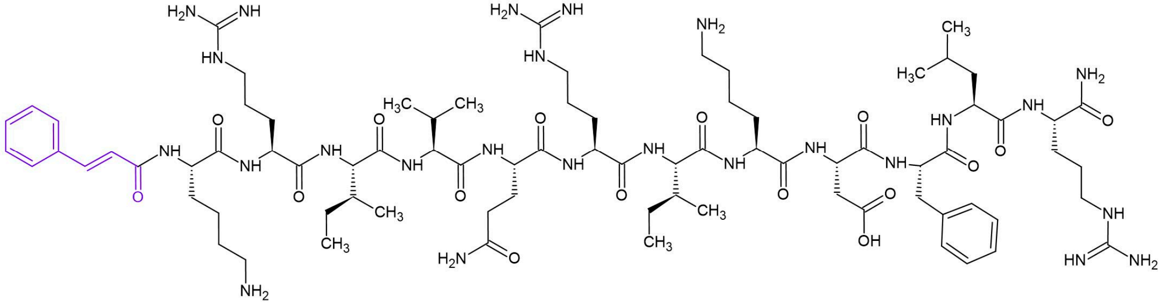
X	KR12-NH_2	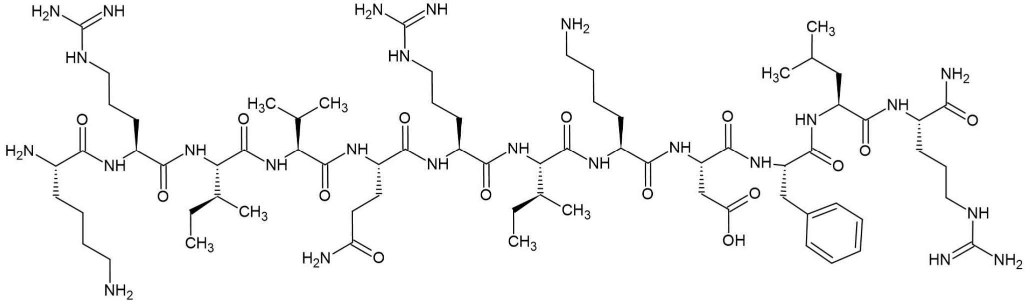
XI	C_8^{ε}-KR12-NH_2	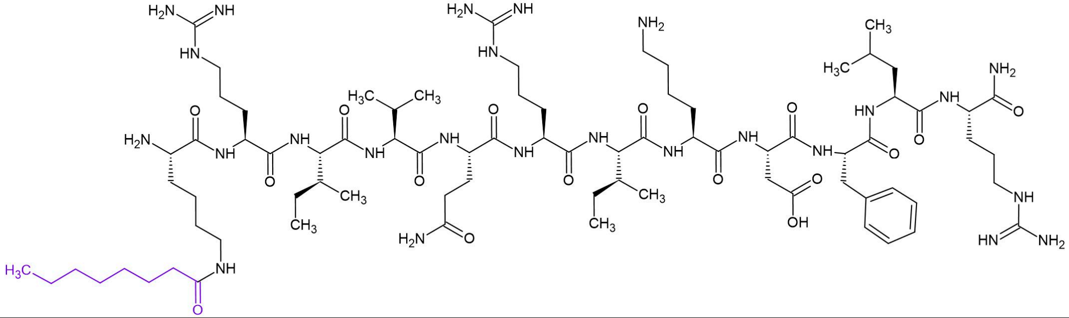

XII	C_8^{α}-Lys-KR12-NH_2	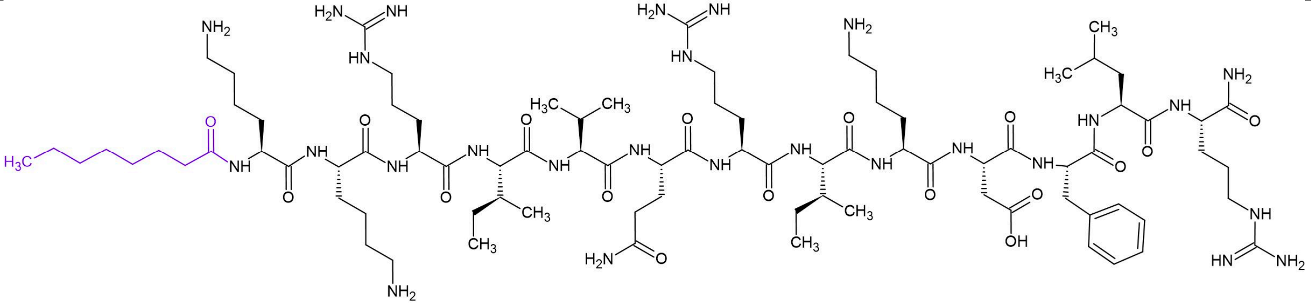
XIII	C_8^{ε}-Lys-KR12-NH_2	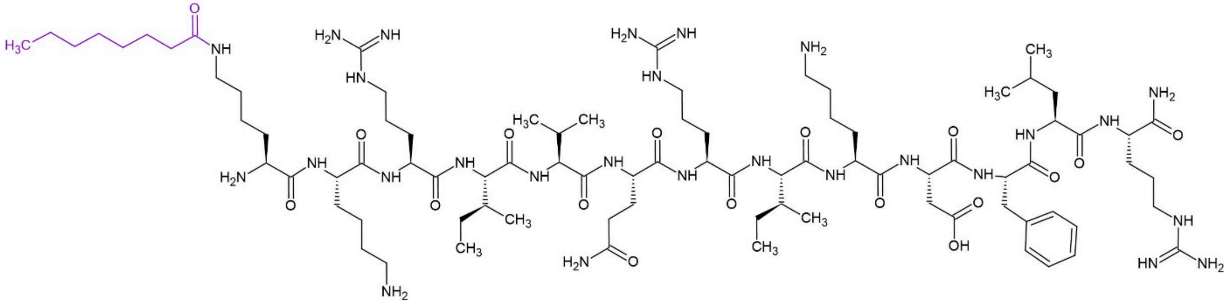
XIV	KR12-$Lys^{\varepsilon}(C_8)$-NH_2	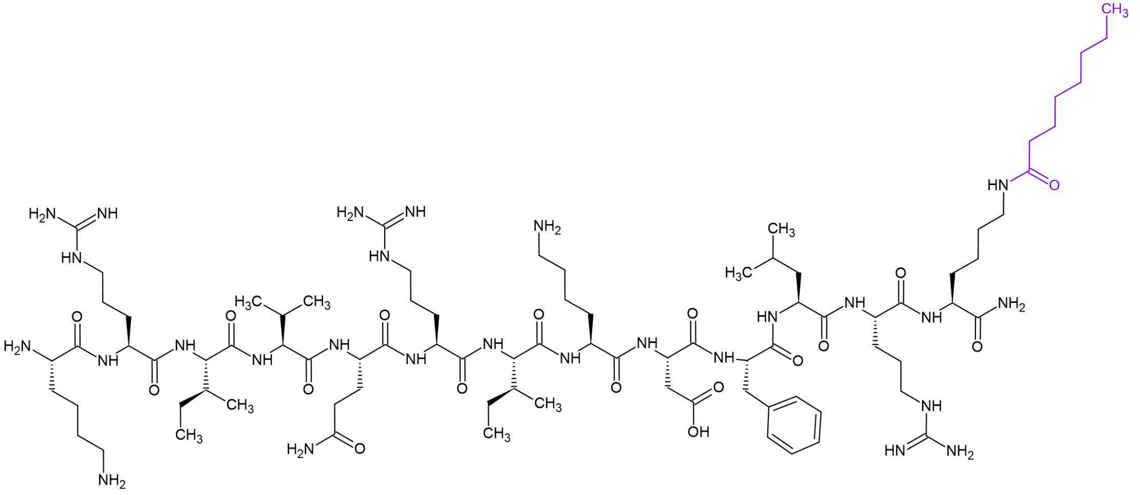

XV	$[Lys^{\varepsilon}(C_8)]^{12}$KR12-$NH_2$	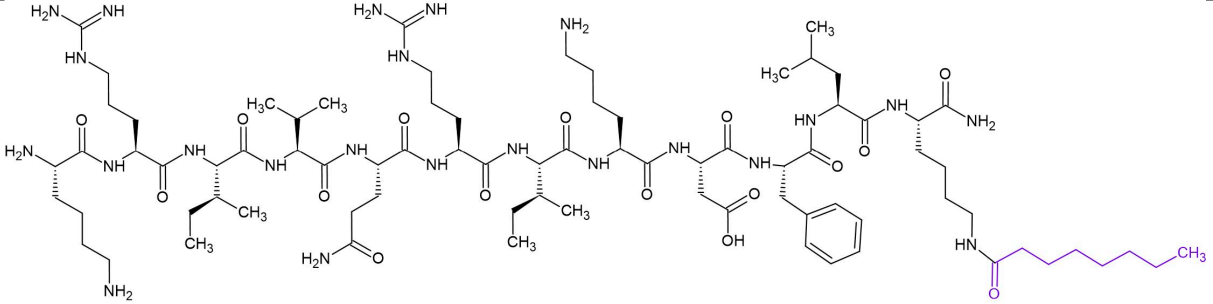
XVI	C_8^{α},C_8^{ε}-KR12-NH_2	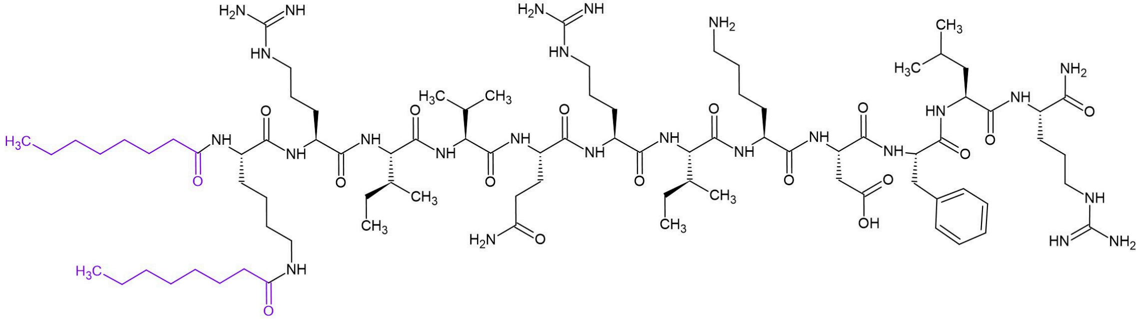
XVII	retro-KR12-C_8^{ε}-NH_2	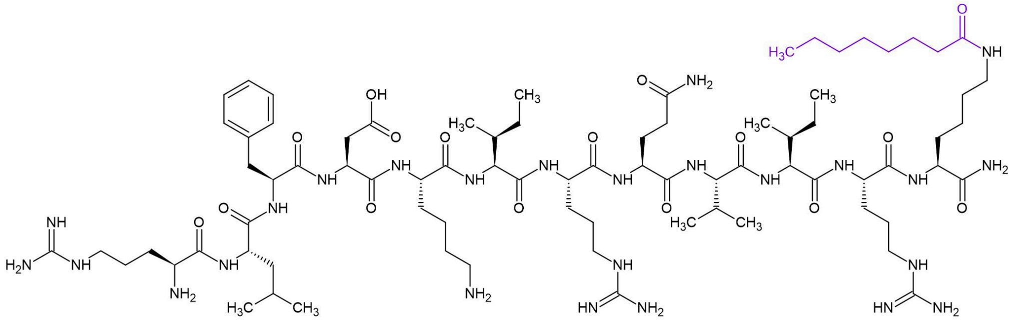

XVIII	C_8^{α}-retro-KR12-NH_2	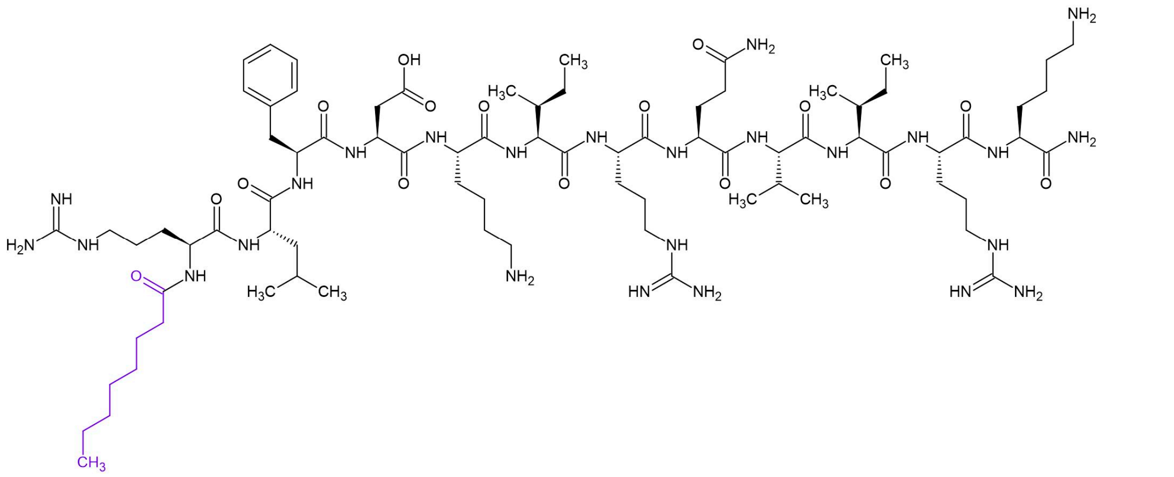
XIX	2-Butyloctanoic acid-KR12-NH_2	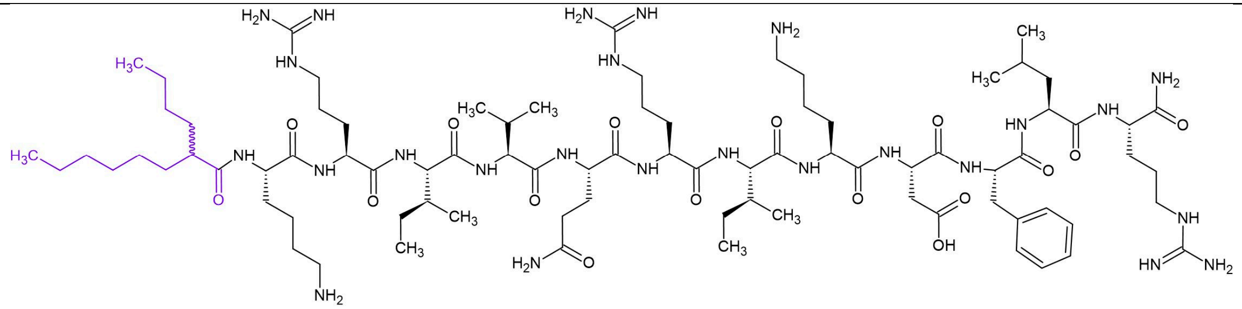
XX	2-Ethylhexanoic acid-KR12-NH_2	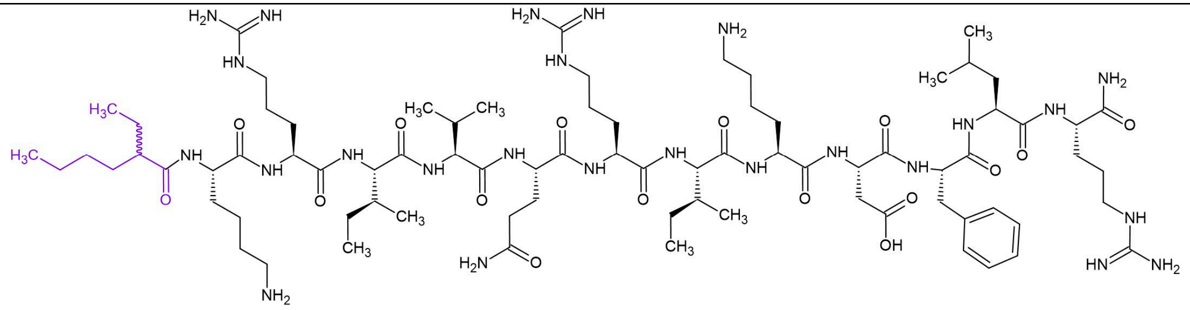

XXI	Phenylacetic acid-KR12-NH2	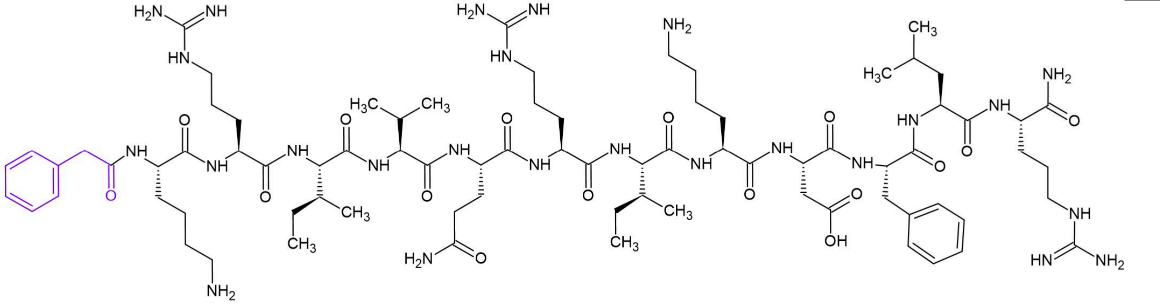
XXII	3-Phenylpropionic acid-KR12-NH2	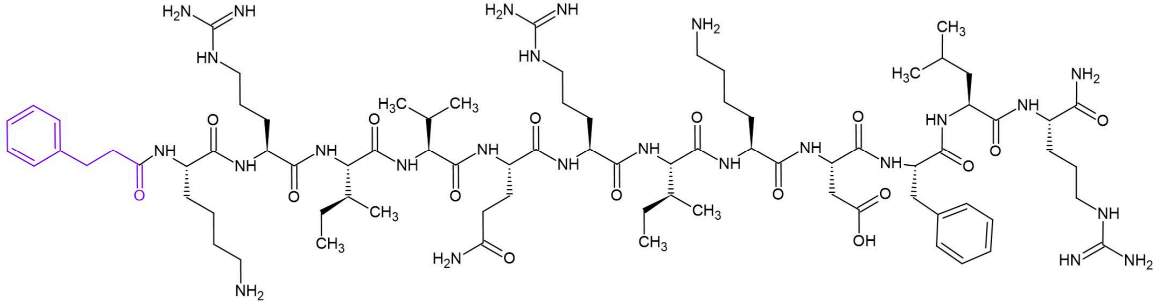
XXIII	4-Phenylbutanoic acid-KR12-NH2	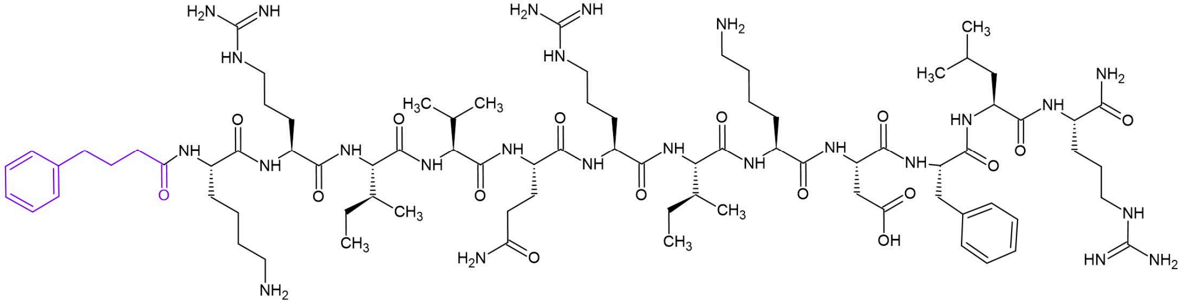
XXIV	Phenylpropiolic acid-KR12-NH2	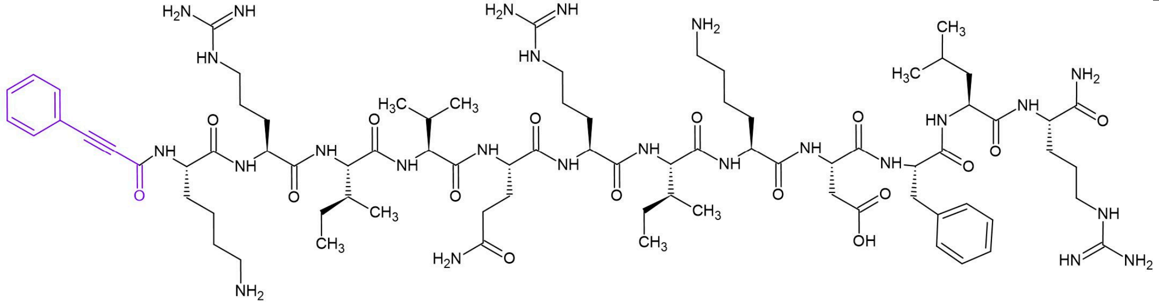

XXV	4-Phenylbenzoic acid-KR12-NH_2	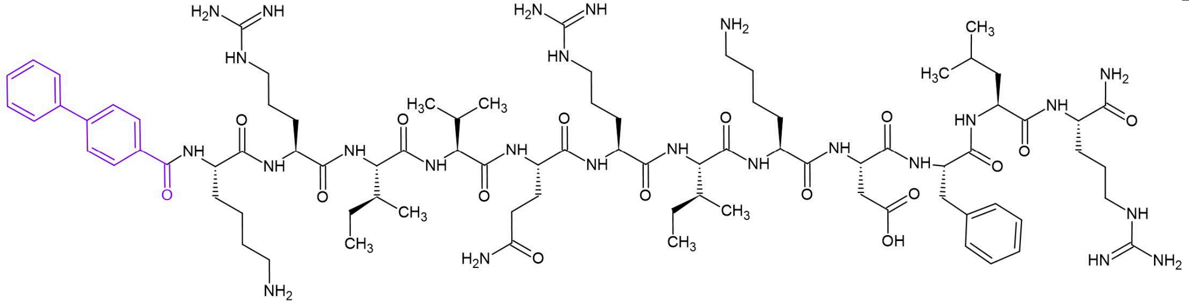
